# The Effect of Differentiated Instruction on the Academic Achievement and Opinions of 3rd-Grade Students in Science Education: A Mixed-Methods Study

**DOI:** 10.3390/jintelligence13100126

**Published:** 2025-10-01

**Authors:** Serpil Kara, Aysun Tekindur

**Affiliations:** 1 Ahmet Keleşoğlu Education Faculty, Necmettin Erbakan University, 42090 Meram, Konya, Türkiye; 2 Ministry of National Education, 06420 Çankaya, Ankara, Türkiye; aysunsenturk86@gmail.com

**Keywords:** differentiated instruction, individual differences, multiple intelligences, different areas of interest, science education, electricity, academic achievement, inclusive teaching, tiered instruction, primary school students, student opinions, mixed method

## Abstract

The purpose of the current study is to determine the effect of the differentiated instruction approach on 3rd-grade primary school students’ academic achievement (N = 45) in the “Electrical Devices and Tools” unit and to explore their opinions regarding the differentiated instruction process. In this context, the content of the lessons prepared using student-centred approaches on students’ science achievement was examined, and students’ opinions on the differentiated instruction approach were also evaluated. The study was conducted in the spring term of the 2024–2025 school year in a major city located in the central region of Türkiye, and a mixed research design combining both quantitative and qualitative approaches was employed. In the current study, during the instructional process of the experimental group, differentiated instruction lesson plans available on the Education Information Network (EIN) portal provided by the Ministry of National Education (MoNE) were used. In the control group, the process outlined by the current curriculum was followed. When the findings were evaluated, statistically significant differences were found in favour of the experimental group, in which activities were implemented based on the differentiated instruction plan, compared to the control group that received instruction within the framework of the current curriculum. In addition, students’ opinions regarding the process indicated that the implementation contributed positively to their learning. In light of the findings obtained, recommendations were made for future research.

## 1. Introduction

### 1.1. Theoretical Framework

Rapid developments in science and technology cause the competencies expected from individuals to change constantly. This change has made the quality of services offered by educational institutions more important than ever for individuals to adapt to the needs of the age. Quality education plays a primary, decisive and key role in achieving sustainable development (UNESCO 2015). In this context, it is accepted that quality education is a strategic element that can direct the development of individuals as well as the international competitiveness of countries (Elcan Kaynak et al. 2023).

In this regard, the importance given to individual differences has increased, and many researchers have emphasized that individuals’ readiness levels, interests, learning styles and needs should be taken into account on the path toward achieving this goal (Heacox 2002; Tomlinson 2014).

Differentiated instruction is also regarded as an inclusive teaching approach, as it allows multiple options to be offered in the learning process by taking into account students’ prior experiences, readiness levels, interests and learning profiles (Estaiteyeh and DeCoito 2023). Differentiated instruction has long been recommended as a pedagogical approach that takes into account individual differences and diversity, particularly in classrooms where students with varying ability levels are present together (Marks et al. 2021).

The focus of differentiated instruction is on students’ individual differences. The instructional process, learning environment and learning products are structured based on these differences (Tomlinson 2014). The main purpose of this approach is to maximize individuals’ knowledge, skills, attitudes and behaviours through an instructional process that is planned and conducted according to each individual’s unique characteristics (Dixon et al. 2014; Tomlinson 2014). Although it is considered a new approach, examples of its application date back to earlier times (Öner 2022). The best example of this is combined classrooms, where students from different grade levels learn together in the same class (K. M. Anderson 2007).

The differentiated instruction approach is grounded in various learning theories. Essentially, it relies on the integration of this theoretical diversity (Gregory and Chapman 2007). According to Gardner’s theory of multiple intelligences, individuals possess eight distinct areas of intelligence: Verbal-linguistic intelligence, logical-mathematical intelligence, visual-spatial intelligence, body-kinaesthetic intelligence, musical-rhythmic intelligence, interpersonal (social) intelligence, intrapersonal (self) intelligence and naturalistic intelligence (Gardner 1999). The dominant intelligence area can vary for each student. In other words, each student has different strengths in thinking and learning. Students learn and develop more easily when they use their strengths. For this reason, an instructional process planned by focusing on only one area of intelligence may limit the learning and development of individuals who are not strong in that area. At this point, differentiated instruction takes into account that each student in the classroom has strengths in different areas of intelligence. Accordingly, the teacher plans lessons not with a single strategy, but enriches them at times with visuals, music or group work. With teaching methods and practices that cater to individual differences, students express themselves more comfortably, become more motivated to learn and their learning becomes more meaningful (Gardner 1999; Gregory and Chapman 2007). According to the constructivist approach, students are expected to take an active role in the instructional process and take responsibility for their own learning. To do so, students should know themselves, become aware of their learning styles, interests and abilities, and achieve instructional goals at their own learning pace (Wibowo et al. 2025). Piaget’s cognitive development theory argues that each individual’s learning style differs from others depending on their developmental level (Gregory and Chapman 2007). Vygotsky’s Zone of Proximal Development (ZPD) theory (Vygotsky 1978) focuses on the gap between the student’s current level and the target level they are expected to reach. It argues that instruction should be conducted slightly above the student’s current level. In this context, it provides a strong framework for differentiation by allowing each student to go beyond their own limits (Gregory and Chapman 2007). Brain-based learning approaches emphasize that lasting and effective learning cannot be achieved in learning environments where students’ fundamental neuropsychological needs, such as emotional security, interest and meaning-making, are not met (Jensen 2008; Sousa 2017; Gregory and Chapman 2007). Kolb’s experiential learning theory highlights the differences between the ways individuals perceive and process information. According to this theory, effective instruction requires strategies to be determined by considering students’ learning styles (Gregory and Chapman 2007; Kolb 1984).

The concept of learning styles is associated with various theories that aim to explain learning differences among individuals and how they acquire knowledge (Hattie and O’Leary 2025). On the other hand, some critics argue that these approaches are responsible for creating an intellectual hierarchy among learning methods aligned with intelligence, placing visual learning at the top and kinaesthetic learning at the bottom (Fallace 2023). Hattie and O’Leary (2025), who examined the main claims regarding learning styles, reveal that this concept has been supported by many different theories over time and defined in various ways. Fallace (2023), who addresses the historical origins of these fundamental claims, draws attention to the changes and transformations in concepts such as learning methods, learning preferences, thinking styles, mind styles and cognitive styles, along with the definition and use of learning styles. According to Hattie and O’Leary (2025), these concepts are still evolving, and one of the main claims in the literature is that learning styles are based on the categories of visual, auditory, kinaesthetic and reading/writing; however, the meanings of these categories have undergone significant changes over time. 

Zaier and Maina (2022) state that teachers can implement differentiated instruction by adapting lesson content to students’ individual learning styles or by grouping them according to shared interests, topics and abilities. Hachfeld and Lazarides (2021), on the other hand, state that in this process, various instructional strategies such as forming homogeneous or heterogeneous groups based on students’ interests and performance and assigning tiered tasks can be effectively employed. The differentiated instruction approach, supported by various learning theories, provides an inclusive learning environment where each student can develop in ways that suit them (Kavut 2024).

The theoretical framework of the current study is grounded on the principles of the constructivist approach by focusing on students’ active participation, meaning-making, and learning through experience. Gardner’s Multiple Intelligences Theory is functional in explaining individual differences, which form the basis of differentiated instruction. While this theory suggests that learners can access knowledge in different ways according to their learning preferences, instructional strategies are teaching practices designed with these preferences in mind. In this context, although learning preferences and instructional strategies are distinct concepts, they have a complementary relationship within the framework of the differentiated instruction approach. Experiential and brain-based learning theories, on the other hand, emphasize the emotional, physical and cognitive engagement of students in the learning process and support the flexibility of the strategies used based on multiple intelligences. Differentiated instruction is defined as adaptations in content, process, product and learning environment according to students’ readiness levels, interests and learning preferences (Tomlinson 2014). The relevant literature also supports that this instructional strategy directly aligns with Gardner’s Multiple Intelligences Theory, which is based on the assumption that not every student learns in the same way (Akpan 2025; Kaziya 2025; Murdin et al. 2025; Subhashini 2025). 

In the current study, the differentiated lesson plans that form the basis of the intervention were designed based on learning theories such as multiple intelligences, constructivism and brain-based learning. Each theory provided some kind of guidance on how the lesson plans should be structured in terms of content, process and product. 

For example, since the multiple intelligences theory requires addressing students’ different learning styles and strengths, the lesson plans included visual, auditory, kinaesthetic and verbal activities. In this way, each student was able to actively participate in the lesson according to their dominant type of intelligence. 

Based on the constructivist approach, students were encouraged to connect new information with their prior knowledge and actively construct the new information. In this connection, a pre-test was administered to assess students’ prior knowledge of the subject and in groups adjusted according to their individual levels, gaps in prior knowledge were addressed step by step, facilitating the construction of concepts related to the subject. In addition, student-centred activities such as open-ended questions, problem-solving tasks and group work were preferred in the lesson plans. 

Moreover, in line with the principles of brain-based learning, the learning environments were designed to engage the senses, stimulate curiosity and provide an emotionally safe setting. Product-oriented activities such as argumentation, drama, posters and poetry used in the plans were aimed at supporting the brain’s learning with multiple stimuli and strengthening permanent learning by activating the affective, cognitive and social domains simultaneously. 

In this context, the theoretical foundations directly guided the intervention design, and the differentiated lesson plans were used as a tool to concretize the applicability of the relevant theories. 

Differentiated instruction is an approach in which the content, process, product dimensions of curricula or lesson plans, as well as the learning environment, are structured by taking into account students’ readiness levels, needs, interests and learning styles. This approach supports each individual’s learning at their own pace and style by offering students various ways to acquire knowledge, apply it and produce outcomes. Thus, the goal is for all students to effectively learn the same concepts, even if through different methods (Heacox 2002; Tomlinson 2014). In this approach, students’ characteristics guide a specific dimension of instruction. This situation provides teachers with a holistic perspective in determining what and how to teach, what to expect from students as learning outcomes and how to organize the learning environment:

- Differentiation based on students’ readiness levels generally takes place in the content dimension. Content is differentiated by selecting texts, materials or activities that are appropriate for students’ current knowledge, skill levels and learning backgrounds (Koeze 2007; Tomlinson 2014).
- Differentiation based on learning styles is mostly oriented toward the process. The learning process is individualized for students having stronger visual, mathematical or verbal/linguistic intelligence through activities such as drawing mind maps, conducting experiments or engaging in discussions (Demir 2021).
- Differentiation based on areas of interest generally takes place in the product dimension. Students can design posters, banners or videos based on their interests or they may have the opportunity to express what they have learned in a personal way through products such as stories or poems (Melesse and Belay 2022; Tomlinson 2014).
- Students’ emotional and environmental needs are important for organizing the learning environment. Options such as the physical features of the classroom, the use of digital tools or group work can be differentiated based on students’ attention levels, motivation and preferences for working individually or collaboratively (Melesse and Belay 2022; Tomlinson 2014). The dimensions of differentiated instruction based on student characteristics and examples of its implementation are presented in Table 1.

In the Science Curriculum, which began to be implemented gradually starting from 2024, differentiated instruction was defined for the first time as one of the core elements of the curriculum under a separate heading (MoNE 2024). Previous curricula did not explicitly mention this approach; instead, there was only an indirect emphasis through general statements addressing individual differences (MoNE 2013, 2018). This development indicates that the curriculum has been transformed into a flexible structure focused on individual differences. The European Commission (2023) argues that, in line with the opportunities offered by new technologies such as digitalization and learning analytics, differentiated instruction should become an integral part of modern curricula. Similarly, the OECD (2018) emphasized that personalized teaching methods should be prioritized in today’s curricula. It considers differentiated instruction as one of the cornerstones of an inclusive and equitable education approach. The PIKTES (Promoting Integration of Syrian Kids into the Turkish Education System) program, supported by the European Union, aims to actively involve foreign-national students living in Türkiye in the education system (PIKTES 2024). It carries out various supportive initiatives to help disadvantaged students benefit from educational opportunities. In schools where PIKTES (2024) is implemented, there are students who differ from each other in terms of language, religion, culture and previous learning experiences. This situation makes it necessary to take different individual characteristics into account during the teaching process. Differentiated instruction plays an important role in the sustainability and effective implementation of such programs. Inclusive education programs like PIKTES emphasize that differentiated instruction is not only an educational approach but also a philosophy based on equity of opportunity in education.

### 1.2. Review of the Previous Studies

A review of the relevant literature reveals that there are many studies showing that differentiated instruction has positive effects on increasing the academic achievement of students in different disciplines such as science, mathematics, Turkish and social studies (Avcı et al. 2022; Kaplan 2016; Koeze 2007; Luster 2008; Prast et al. 2018; Salar 2018; Suprayogi et al. 2024; Swift 2009; Taş and Minaz 2024) and on developing various higher-order skills such as scientific process skills, critical thinking and problem solving skills (Al-Shehri 2020; Anggareni and Hidayat 2022; Avcı et al. 2022; Kaplan 2016; Titus 2025; Yabaş and Altun 2009). When the literature is generally evaluated, it is seen that most studies on differentiated instruction are conducted using experimental methods, followed by survey and mixed-method studies. In data collection, achievement tests, scales, interviews and observation forms have been predominantly used (Avcı et al. 2022). In addition, it is notable that differentiated instruction practices are particularly concentrated in studies conducted on gifted students (Ardenlid et al. 2025).

In a study conducted by Smale-Jacobse et al. (2019), the results of fourteen different studies were systematically reviewed to evaluate the effect of differentiated instruction on the academic achievement of secondary school students. The findings showed that differentiated instruction has a positive effect of small to moderate effect size on the academic achievement of secondary school students. Suprayogi et al. (2024) reported that differentiated instruction has a significantly positive effect on the science achievement of elementary school students. Research results emphasize that differentiated instruction is an effective approach in science teaching. In a study conducted by Anggoro et al. (2024), multiple intelligence-based differentiated instruction activities were implemented at the elementary school level. It was determined that through applications based on this approach, students were able to maximize their verbal, mathematical, body-kinaesthetic, social and intrapersonal intelligences. Moreover, it was reported that significant improvement occurred in students’ levels of recalling, understanding and applying science concepts. In a study examining the effectiveness of differentiated instruction in improving students’ academic achievement and higher-order thinking skills, it was reported that students who received differentiated instruction showed significant improvement in higher-order thinking skills such as analysis, evaluation and creation (Balita and Salvador 2023). In a different study examining the effect of differentiated instruction on mathematics achievement in elementary school, classroom practices of 24 teachers in the Netherlands were observed, and student achievement was measured using a mathematics test. The results showed that differentiated instruction increased achievement; however, this increase was not statistically significant. The study concluded that the inability of teachers to provide sufficient variety to address individual differences during the differentiation process, as well as limitations related to the assessment tool, may have influenced the results (Mulder 2014). Another study examining teachers’ differentiated instruction practices indicated that these practices were generally carried out by considering a limited number of student characteristics. It was especially emphasized that the measurement and evaluation processes lack the flexibility and individuality required by differentiated instruction (Bekler and Kozikoğlu 2024). In the literature, various studies examining teachers’ practices based on the differentiated instruction approach are frequently encountered (Çam and Acat 2023; Griful Freixenet et al. 2020; Melesse 2016; Mengistie 2020; Mutlu and Öztürk 2017; Pozas et al. 2020; Yenmez and Özpınar 2017; Zoraloğlu and Şahin 2022).

When the results of relevant studies in the literature are generally evaluated, it is seen that practices based on differentiated instruction are an effective method for increasing students’ academic achievement, motivation and higher-order cognitive skills (Avcı et al. 2022). 

### 1.3. Rationale and Significance of the Study

Differentiated instruction, by its general nature, is an educational approach that is sensitive to individual differences and based on inclusivity and equity of opportunity (OECD 2018). Primary school students are in a rapid and variable period of both cognitive and affective development (Rabindran and Madanagopal 2020). Therefore, differentiated instruction applied to this age group will contribute to the development of positive attitudes toward learning by responding to individual needs at an early stage. Science lessons are often found difficult to understand due to the presence of mostly abstract concepts (Gilbert 2004). This abstractness can lead to students acquiring misconceptions or developing alternative conceptions (Yavuz and Akça 2020). In this context, it is stated that there are particular difficulties in teaching the electricity unit, one of the science topics, especially at the primary school level (Carlton 1999; Kalogiannakis et al. 2018). At this educational level, where the foundations of knowledge, skills and attitudes related to science are established, the accurate and deep understanding of concepts is important not only for supporting academic achievement but also for increasing students’ interest and participation in scientific activities. Based on these needs, this study aims to evaluate the effect of the differentiated instruction approach used in the teaching of the “Electrical Devices and Tools” unit on the academic achievement of 3rd-grade primary school students, as well as to evaluate the students’ opinions regarding the process. 

In the study, differentiated instruction activities prepared by taking into account the students’ readiness levels and areas of interest were implemented. Students’ readiness level is related not only to their prior knowledge of the subject but also to their cognitive development, learning pace and comprehension skills (L. W. Anderson 1991). The current study makes a unique contribution to the literature by implementing differentiated instruction activities in science classes at the primary school level, specifically focusing on the “Electrical Devices and Tools” unit. Although there are similar studies in the literature on differentiated instruction practices for science classes, these studies are particularly limited in number at the primary school level and they do not include students’ opinions regarding the process. The current study aims to fill an important gap at both national and international levels by not only evaluating academic achievement but also revealing students’ opinions. For students with different learning profiles, it is expected to provide a supportive contribution to academic achievement in science lessons where abstract concepts are taught. Moreover, the current study provides teachers with concrete and practical examples of how the differentiated instruction approach can be effectively implemented in the classroom, especially for early age groups where individual differences based on cognitive development are prominent. The findings obtained will provide guidance to curriculum developers regarding the effects of this approach on primary school students. 

The main problem and sub-problems posed in the current study to determine the effects of differentiated instruction on primary school students are as follows: 

### 1.4. Research Problem

What is the effect of the differentiated instruction approach used in the teaching of the unit “Electrical Devices and Tools” to 3rd-grade students on their academic achievement? Moreover, what are the participants’ opinions on this instructional process? 

#### Sub-Problems

Sub-problem 1: Is there a significant difference between the pre-test academic achievement scores of the experimental and control group students?

**H0_1_:**  *There is no significant difference between the pre-test achievement scores of the experimental and control group students.*

**H1_1_:**  *There is a significant difference between the pre-test achievement scores of the experimental and control group students.*

Sub-problem 2: At the end of the intervention, is there a significant difference between the post-test academic achievement scores of the experimental and control group students?

**H0_2_:**  *There is no significant difference between the post-test achievement scores of the experimental and control group students.*

**H1_2_:**  *There is a significant difference between the post-test achievement scores of the experimental and control group students*.

Sub-problem 3: What are the experimental group students’ opinions on the differentiated instruction process? 

## 2. Materials and Methods

In this section, detailed information is provided about the research design, study group, instructional sequence of groups, data collection tools and data analysis.

### 2.1. Design of Research

The current study aimed to determine the effect of using the differentiated instruction approach in the teaching of the “Electrical Devices and Tools” unit on the academic achievement of 3rd-grade primary students, as well as the students’ opinions on the process. In line with the purpose of the study, a mixed-methods research design combining both quantitative and qualitative approaches was employed (Plano Clark and Creswell 2008). A concurrent triangulation design, one of the mixed-methods research designs, was used in the study (Creswell 2003). Using two or more complementary and integrated methods together in a study, rather than relying on a single method, is an approach aimed at providing depth and variety in the methodology. This approach involves the simultaneous and purpose-driven use of both quantitative (such as surveys and scales) and qualitative (such as interviews, observations and document analysis) data collection techniques within the same study, especially during the data collection process (Morse 1991). In this regard, quantitative and qualitative data were collected and analyzed simultaneously but independently of each other. The findings obtained were compared at the end of the process to examine whether the two types of data supported each other (Creswell 2014). 

In the quantitative dimension of the study, a pre-test-post-test matched control group quasi experimental design was used. The study groups were determined by matching pre-existing groups on certain variables without random assignment (Fraenkel et al. 2012). Table 2 summarizes the experimental design of the study. 

**Figure 1:**
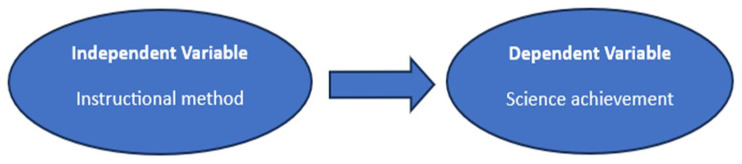
Variables of the study.

In the experimental design of the study, science achievement was taken as the dependent variable. The independent variable whose effect on this variable was examined is the instructional method used. The independent and dependent variables of the study are visualized in Figure 1. 

In the qualitative dimension, two students from each of the high, medium and low achievement levels were selected to reveal their opinions on the differentiated instruction practices, and semi-structured interviews were conducted with a total of six students. This selection is based on the purposive sampling strategy, which is commonly used in qualitative research. Purposive sampling allows for the inclusion of individuals who can provide rich information, thereby adding depth to the study (Palinkas et al. 2015). In this context, selecting students with strong verbal-linguistic skills made it possible to obtain more comprehensive and detailed data regarding the effectiveness of the intervention. 

In the literature, it is stated that in qualitative research, emphasis is placed more on the diversity of the data obtained and achieving data saturation rather than on the sample size. One of the most commonly cited criteria for justifying sample size has been shown to be saturation (Vasileiou et al. 2018). Hennink and Kaiser (2022) argue that even 4–8 interviews may be sufficient to reach thematic saturation. Similarly, Squire et al. (2024) showed that in many studies, core themes begin to recur at early stages, indicating that saturation can be achieved at this point, and that conducting additional interviews often reduces time and resource efficiency. The data obtained from the interviews were analyzed using the content analysis method. A quasi-experimental design with experimental and control groups was used in the quantitative dimension, while a semi-structured interview form was employed in the qualitative dimension, and content analysis was conducted. The main purpose of content analysis is to group data with similar characteristics around specific concepts and themes and then organize and interpret these data in a way that is easily understandable to the reader (Yıldırım and Şimşek 2011). In the current study, a coding approach based on concepts derived from the data was adopted during the data coding process, as recommended by Strauss and Corbin (1990). The process followed involved creating codes, identifying themes, systematically organizing the codes and themes and finally describing and interpreting the findings obtained (Yıldırım and Şimşek 2011). 

Since the study was conducted at a school of the Ministry of National Education and involved primary school students, ethical approval was first obtained from the ethics committee of the university to which one of the researchers is affiliated. Following the ethical committee approval, permission to conduct the research at the school was requested by another researcher through an application to the relevant provincial directorate of national education. After obtaining the necessary ethical and administrative permissions, the study was initiated.

Information about the ethical approval obtained for this study is as follows. The study was conducted with ethical approval obtained from the Social and Human Sciences Ethics Committee of Necmettin Erbakan University, dated 18 April 2025, decision number 2025/327, and the meeting number was 8. Then, the research permission was approved by the Provincial Directorate of National Education of the province where the study would be conducted, with application number MEB.TT.2025.025020 and the institution code of 709621. 

### 2.2. Study Group

The study group of the current study was determined during the spring term of the 2024–2025 school year. The study group consists of 3rd-grade students attending a public primary school in a district of a large province located in the Central Anatolia Region of Türkiye. 

There are a total of 280 third-grade students studying at the school. Two classes were randomly selected from the third-grade classes in this school and one of them was assigned as the experimental group (N = 24) and the other as the control group (N = 21). The reason for selecting this study group was that one of the researchers was working at the same school. This allowed the process to be monitored more effectively, the data to be collected more reliably and the instructional process to be directly intervened in a controlled manner. 

In the study, in order to minimize the impact of teacher and student differences in the groups on the research results, the classes to participate were determined through matching, taking certain variables into account. In this context, the participating teachers were matched in terms of age, undergraduate program completed and professional experience. Then, the first-term report card grades of the 3rd-grade science course for the students in the experimental and control groups were examined, and it was evaluated whether there was similarity between the groups in terms of achievement levels. After it was observed that there was no significant difference between the groups in terms of the teacher and student variables, pre-tests were administered, and the results of these tests also showed no statistically significant difference between the groups. Based on these data, the experimental and control groups were determined. 

The information about the teachers in the study groups is presented in Table 3.

The information regarding the first-term report card grades in the 3rd-grade science course for the students in the study groups is presented in Table 4. 

### 2.3. Instructional Sequence of Groups

A pre-test was administered to both the experimental and control groups during the first week. The implementations, including the pre-test and post-test administrations, were completed within a total period of 5 weeks. The implementations carried out over the five-week period can be summarized as follows:

In the first week, a pre-test was administered to both the experimental and control groups. After the pre-test was administered, the experimental group was divided into three levels based on the test results, and preparations were made for the activities to be conducted for each level. These preparations included producing the necessary activity materials as printouts and ensuring that the teacher obtained and prepared other required materials according to the lesson plan to be implemented.

The 2nd, 3rd and 4th weeks were devoted to the applications. The lessons in the experimental group were conducted in accordance with the differentiated instruction approach by a teacher who is also one of the researchers and currently serves as the primary teacher. This teacher participated as a participant in the “Addressing Learning Losses and Strengthening Instruction in Basic Education” project, conducted in collaboration with the Ministry of National Education and UNICEF during the 2024–2025 school year. Within the scope of this project, the teacher underwent a comprehensive training process covering the theoretical foundations of differentiated instruction and its implementation steps. Moreover, the teacher undertook an active role in preparing lesson activities based on differentiated instruction during the project. The lessons in the control group were conducted over three weeks using the teacher’s own established instructional methods, in accordance with the methods and processes prescribed by the existing curriculum, without any external intervention. To assess whether any factors that could affect the validity and reliability of the research results had emerged, a direct observation of one lesson in the control group was conducted. This observation was conducted by the researcher in an unstructured (natural) manner, and no guidance was provided or no intervention was made throughout the process. In this observation, it was determined that the teacher primarily took on the role of information conveyor, while the students participated mostly as listeners in the process. 

In the lessons of the control group, the teacher predominantly used the lecturing, question-and-answer and demonstration methods. In the lessons, instructional materials such as the science textbook and smart board were frequently used. The subjects covered were first explained by the teacher in accordance with the content in the textbook, and then the activities in the textbook were carried out by the class. The question-and-answer method was used to assess students’ prior knowledge and to check their learning. The content presented by the teacher was supported with visual and auditory materials using various educational portals through the smart board. In addition, the teacher occasionally posed thought-provoking questions during the lesson to create discussion opportunities related to the subject within the classroom. Throughout the process, students worked individually, and no group work was conducted. 

In the current study, during the instructional process for the experimental group, differentiated instruction lesson plans available on the Education Information Network (EIN) portal provided by the Ministry of National Education (MoNE) were utilized. 

MoNE defines the purpose of EIN as enabling the use of information and communication technologies and educational materials in any environment, regardless of location, and integrating technology into the teaching process (EIN 2020). The e-content available on EIN offers rich and inclusive materials that cater to students with different learning profiles, such as verbal, visual, auditory, numerical, intrapersonal and social (Gürsoy and Uğurlu 2018). There is also a separate section on the EIN portal that students can access and benefit from. Differentiated instruction lesson plans are located in a special section on the EIN portal that is accessible only to teachers and serve as a guide resource aimed at enriching the teaching process. 

#### Intervention Process

The implementations carried out in the experimental group can be summarized as follows:

Before the intervention, the students in the experimental group were divided into three different groups based on their prior knowledge, in other words, their readiness levels, regarding the “Electrical Devices and Tools” unit. For this purpose, the students’ pre-test scores from the Electrical Devices and Tools – Academic Achievement Test (EDT-AAT) were used. Based on the scores they received from the test, the students were divided into the following groups:

- Group A (0–9 points): Students have insufficient prior knowledge about the unit and possess a low level of readiness regarding the current learning outcomes.
- Group B (10–13 points): Students have borderline prior knowledge about the unit and possess a moderate level of readiness regarding the current learning outcomes.
- Group C (14–20 points): Students have sufficient prior knowledge about the unit and are ready for the current learning outcomes.

Accordingly, 9 students were included in Group A, 11 students in Group B and 4 students in Group C. Group A continued their work divided into two groups of 5 and 4 students, Group B worked as two groups of 6 and 5 students and Group C worked as a single group of 4 students. 

In the student groups formed based on readiness levels, the teaching process was conducted using lesson plans prepared according to the tiered teaching strategy of the differentiated instruction approach, which are available to teachers on the EIN platform. In the tiered teaching method, the content, process and product dimensions of the instruction are differentiated by considering students’ readiness levels, learning speeds and cognitive abilities. Each student achieves the same learning outcome through activities tailored to their own level and pace. Before starting this process, students’ prior knowledge is determined (Demir 2021).

The students worked in groups but mostly completed the activity sheets individually. During this process, the teacher guided all the groups and directed the flow of the process. In order to concretize the implementation of the instructional process, a sample lesson plan covering three class periods on the topic of “safe use of electricity” was presented: 

During one class hour, the students worked on tasks differentiated according to their readiness levels. The students in Group A engaged in activities aimed at completing the necessary prior learning before moving on to the current learning outcome. Activities based on prior knowledge were prepared by considering the relevant learning outcomes from the previous grade level and the prerequisite concepts of the current learning outcome as outlined in the curriculum. During this process, Group A conducted group discussions on a concept cartoon related to emergencies caused by electrical devices. In this activity, misconceptions about electric shock were addressed by distinguishing between correct and incorrect statements. In addition, a scenario-based drama was used to demonstrate which authorities to seek help from and how, in the event of a fire caused by electricity.

The students in Group B, having a moderate level of readiness, first reinforced their learning with a brief prior knowledge activity related to the subject. This stage was not as basic as it was for the students in Group A. It primarily served to review existing knowledge and prepare for the learning outcome. During the prior knowledge stage, the students were given short scenarios regarding the correct and incorrect use of electrical devices and through discussions of these scenarios, they identified the proper practices. Then, the students worked on visuals depicting situations in which electrical devices are not used safely. They discussed the potential dangers that can be caused by the situations depicted in the visuals and reasoned about the necessary safety precautions. Finally, they were asked to write a letter to the individuals depicted in the visuals, explaining the potential dangers and the safety precautions that should be taken. 

The students in Group C, on the other hand, carried out an enrichment activity related to the current learning outcome. They worked on problem situations related to the safe use of electrical appliances, identified the issues and developed solution suggestions. They composed a song, wrote a poem or created a slogan related to the problems they identified and their proposed solutions.

In the next lesson, the students in Group A, after completing their prior learning, participated in the main activities related to the current learning outcome. The students in this group examined simple scenarios taken from daily life and, by identifying the correct behaviours in these situations, created safety usage guidelines. The students in Group B carried out enrichment activities to reinforce the current learning outcome. They expressed the rules for the safe use of electricity through posters and displayed these posters on the classroom walls. The students in Group C, on the other hand, worked on more complex problem situations and developed creative suggestions for solutions. During this process, the argumentation method was used. The students presented suggestions as solutions to the identified problem situation. They supported their claims with justifications and evidence. Then, by comparing their claims regarding different solutions, they conducted discussions on which option is safer and more feasible. Thus, the students not only generated solutions but also developed their skills in defending their ideas, questioning others’ opinions and evaluating alternative viewpoints.

After the activities related to the subject were completed, in the third lesson, the same worksheet was distributed to each group. With the activity on this worksheet, each group shared what they had learned about the same subject with the class. In this way, the groups came together in a common task in accordance with the tiered teaching strategy. This activity process aimed to share and reinforce what had been learned. 

Although the groups were formed based on their readiness levels, there was no strict restriction during the teaching process. Students who completed their own activity were able to participate in the activities of other groups if they wished, thus carrying out the process in a manner consistent with flexibility, one of the fundamental principles of differentiation (Tomlinson 2014).

The division of the students into Groups A, B and C according to their different readiness levels aims, in line with Vygotsky’s Zone of Proximal Development theory (1978), to provide support just above each student’s current level. The argumentation, discussion, drama, and problem-solving activities carried out in the groups ensured students’ active participation and allowed them to construct knowledge based on their own experiences, as envisaged by the constructivist approach (Wibowo et al. 2025). In addition, creating different products such as posters, songs, letters and slogans allowed students to reflect their diverse strengths in the learning process, in line with Gardner’s (1999) multiple intelligences theory.

**Figure 2:**
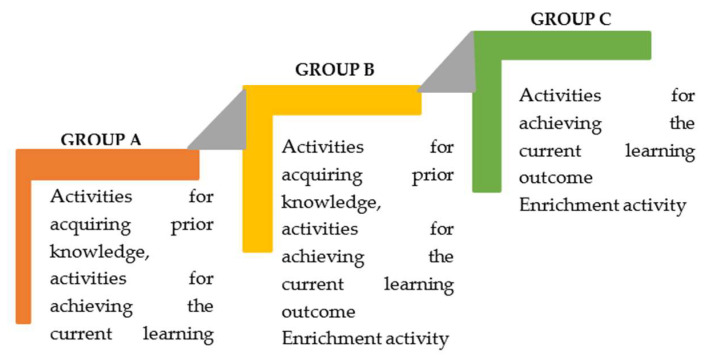
Summary of the implementation process.

A summary of the process related to the aforementioned applications is presented in Figure 2, and information regarding the differentiation process is provided in Table 5.

The new primary school science curriculum, which began to be implemented gradually starting from the 2024–2025 school year, is currently only being applied at the 1st grade level. During the period when the current study was conducted, 3rd grade students were being educated according to the old curriculum. The implementation process for the “Electrical Devices and Tools” unit in the experimental group was planned based on the new science curriculum. However, both curricula commonly included the topics of “characteristics of electrical devices and tools” and “safe use of electricity”. To ensure the integrity and reliability of the study, the teacher in the control group was informed and asked to conduct teaching activities related to the topic of “economical use of electricity” and its learning outcomes, which were not included in the old curriculum. Consequently, both groups conducted activities related to the same topic and learning outcomes within the same timeframe. No external intervention was made in the teaching process of the control group.

In the 5th week, after the implementations were completed, the academic achievement test was administered as a post-test. Then, within the same week, semi-structured interviews were conducted with 6 students from the experimental group who had low, medium and high academic achievement and strong verbal expression skills in order to elicit their opinions on the process. 

### 2.4. Data Collection Tools

In the study, quantitative data were collected using the EDT-AAT, which was prepared by the researchers and whose validity and reliability were tested through pilot applications. Qualitative data, on the other hand, were obtained through the analysis of the semi-structured interviews conducted with students. 

#### 2.4.1. Electrical Devices and Tools-Academic Achievement Test (EDT-AAT)

Within the theoretical and conceptual framework of the study, relevant literature, exam questions prepared by teachers and achievement test questions were extensively reviewed, and a pool of multiple-choice questions for the relevant unit was created. Subsequently, questions targeting the learning outcomes set in the science curriculum were selected. After the necessary adjustments, the EDT-AAT was created as a multiple-choice test consisting of 21 questions. 

For the validity and reliability analyses of the test, pilot applications were conducted with 100 students. One item with low discrimination was removed, and the EDT-AAT was finalized as a 20-question test. Below, Table 6 presents the item analysis results of the EDT-AAT.

In achievement tests, items are expected to be of medium difficulty. This means that the item difficulty value should be around .50 (Atılgan 2017). Based on this criterion, it is understood that the vast majority of the items are of medium difficulty. 

For questions to differentiate between those who know and those who do not, the item discrimination index should ideally be close to +1. If an item’s discrimination index is negative or below .19, it is considered weak and is recommended to be removed from the test. However, items with a value above .30 have strong discrimination and can be used without modification (Tekin 2010). When the item discrimination indexes (r) are evaluated, it is seen that the 10th item in Table 3 has a negative discrimination value (−.07). This shows that the item separates successful and unsuccessful students in the opposite direction. For this reason, it was decided to remove the item from the test. As a result, the number of questions in the achievement test, which initially consisted of 21 questions, was reduced to 20, and the Cronbach’s alpha value of the test was calculated to be .76. This value indicates that the internal consistency of the test is at a good level and the measurement results obtained from the test are reliable (Can 2014).

#### 2.4.2. Semi-Structured Interview Form

To determine the experimental group students’ opinions on the process, semi-structured interviews were conducted with a total of six students across low, medium and high achievement levels and the interview data were subjected to analysis. The opinions of the students selected from the experimental group regarding the process were gathered using a semi-structured interview form and then evaluated. 

The questions in the semi-structured interview form were prepared by the researchers. The interview form was finalized after receiving feedback from academics specialized in science education, argumentation, scientific reasoning and differentiated instruction. The first three questions aim to warm up the students to the interview process and thus to obtain more reliable data. The questions in the semi-structured interview form are as follows:

1. Can you briefly introduce yourself? (How old are you, do you have any siblings?)
2. Which profession do you want to choose in the future? Why?
3. What’s your favorite school subject? What makes you like this subject more?
4. For approximately three weeks, your science lessons were planned and implemented in accordance with the differentiated instruction approach.
  - What differences were there in the way science lessons were conducted during this process, compared to your previous science lessons?
  - Considering these differences, how would you generally evaluate the science lessons during this process?
5. What did you like about the differentiated instruction implementation process? What did you find most beneficial for you? Why?
6. Were there things you found difficult to do during the differentiated instruction activities? If so, how did you solve this problem?
7. Did these lessons, taught with the differentiated method, have any effect on your understanding of the topic and your academic achievement in the course? How?
8. How did you feel during the differentiated instruction activities? When comparing them to your previous science lessons, were there any differences in your feelings and thoughts about science class? Can you explain?
9. Would you like differentiated instruction activities to be used in other science units as well? Why?

The interviews were conducted in a semi-structured format, using probe questions appropriate for the students’ age. The questions were supported with concrete examples when necessary and made clearer through sub-questions. In this way, students’ expression of their feelings and thoughts was facilitated. In addition, students with well-developed verbal-linguistic skills were specifically selected for the interviews to elicit more meaningful and detailed responses. For example, the question “comparison with previous science lessons” was, when necessary, posed as “What were you doing in previous lessons, and what did you do differently in this lesson?” Questions related to feelings were supported with more concrete and simple expressions, such as “Was the lesson fun? Did you get bored? Why?” 

### 2.5. Data Analysis

#### 2.5.1. Analysis of the Quantitative Data

For the analysis of the quantitative data, the results obtained from the EDT-AAT were evaluated. The EDT-AAT consisted of a total of 20 questions. Each correctly answered question was scored as 1 point, while incorrect or blank answers were scored as 0. The lowest score to be taken from the EDT-AAT is 0, while the highest score is 20.

Before proceeding with the analysis of the findings from the quantitative data, the normal distribution of the pre-test scores on the EDT-AAT for the students in both the experimental and control groups was checked. Since the number of students in the study groups was less than 35, the results of the Shapiro–Wilk test (Shapiro and Wilk 1965) were examined for normal distribution. The normal distribution results for the pre-test and post-test scores of the experimental and control groups are presented in Table 7.

Based on the findings, the pre-test data were found to have a normal distribution because their *p*-values were greater than 0.05. Furthermore, the skewness and kurtosis values being within the ±2 range support the assumption that the distribution is normal (George and Mallery 2010). In light of these data, an independent samples *t*-test, a parametric test, was used to evaluate whether there was a statistically significant difference between the pre-test scores of the experimental and control groups. 

On the other hand, the examination of the post-test score distributions revealed that the control group’s post-test scores are normally distributed (*p* = .22), but the experimental group’s post-test scores do not show a normal distribution due to being below the .05 significance level (*p* = .04). 

#### 2.5.2. Analysis of the Qualitative Data

In the content analysis used for the analysis of the qualitative data, the coding process proposed by Strauss and Corbin (1990) was followed, and coding was carried out based on the concepts derived from the data. Then, the analysis process continued with the classification of the codes and the identification of categories and themes, and was completed by following the stages of organizing the categories, describing the findings and interpreting them (Yıldırım and Şimşek 2011). To establish reliability, the data obtained from the semi-structured interviews were coded separately by the researchers, and inter-rater reliability was assessed by calculating the percentage of agreement between the evaluators. Disagreements between coders regarding the assignment of each code to the appropriate theme or category were resolved through constructive negotiations within the scope of the “open discussion and consensus” strategy (Schaekermann et al. 2019). The inter-coder agreement percentage was calculated (Agreement percentage = agreement/(agreement + disagreement) × 100) (Miles and Huberman 1994) and was found to be 94%. 

## 3. Results

In this section, the findings are organized and presented according to the research questions. Statistical findings are explained using graphs.

### 3.1. Results of the Independent Samples t-Test Conducted on the Pre-Test Scores

The first research question is to determine whether there is a statistically significant difference between the pre-test academic achievement scores of the experimental and control group students on the topic of electricity. In this connection, since the results of the normality test indicated a normal distribution, an independent samples *t*-test, one of the parametric tests, was conducted. The analysis results are presented in Table 8.

As a result of the independent samples *t*-test conducted to determine whether there was a significant difference between the pre-test scores of the experimental and control groups, it was concluded that there was no statistically significant difference between the two groups (t(43) = −0.75, *p* > .05). In light of these findings, it can be said that the experimental and control groups had similar prior knowledge regarding the “Electrical Devices and Tools” unit before the implementation.

### 3.2. Results of the ANCOVA Test Conducted on the Post-Test Scores

The second research question is to determine whether there is a statistically significant difference between the post-test academic achievement scores of the experimental and control group students on the topic of electricity. Regression slope analysis was conducted to ensure the homogeneity of the data (Table 9). The results of the regression slope analysis were found to be F(1.41) = 0.668, *p* = 0.419, indicating that the *p*-value was greater than 0.05. This finding indicates that the results were not significant and that the two groups were homogeneous at the beginning of the study. Since the experimental and control groups were homogeneous at the beginning of the intervention, applying ANCOVA to reveal the differences in the post-tests was deemed appropriate. Descriptive statistics and ANCOVA were used to determine whether there was a difference between the two groups.

As shown in Table 10, the descriptive statistics reveal differences between the pre-test and post-test scores of the experimental and control groups.

To determine whether the differences between the groups were statistically significant, a one-way ANCOVA was conducted with the pre-test scores controlled as a covariate. According to the results presented in Table 11, after adjusting for the pre-test effect, a significant difference was found between the experimental and control groups. The obtained values, F(1.42) = 10.036, *p* = .003, indicate that this difference is statistically significant. The *p*-value being less than .05 supports the effectiveness of the differentiated instruction intervention.

In addition, the Partial Eta Squared (η^2^) value, which indicates the effect size, was calculated as .193. This value corresponds to a large effect according to Cohen’s (1988) effect size classification and indicates that the independent variable accounts for 19.3% of the total variance in the dependent variable. Based on these findings, the null hypothesis was rejected and the alternative hypothesis was accepted. As a result, it was concluded that the students in the experimental group performed significantly better than their peers in the control group, indicating that differentiated instruction had both a significant and strong effect on learning.

### 3.3. Findings from the Semi-Structured Interviews

For the third research question, interviews were conducted with six students selected from the experimental group who participated in the implementations to discuss the process. The categories and codes derived from the interviews conducted are presented in Table 12.

According to the findings in Table 12, in the comparison of the differentiated science instruction with the traditional science lessons, the topics the students emphasized most were “group work” and “learning while having fun”. The students stated that during this process, they worked more with their peers, felt that the activities were like games and that this made learning more lasting and easier. The second interviewed student (S2) expressed his/her thoughts as follows: “Before, we always used to write in our notebooks, but now we sometimes worked in groups. I did it together with my friends. We played with cards and did different activities. Sometimes it felt like a game. That’s why it stayed in my mind more.” Another student (S4) emphasized learning more easily with the statement, “When I did it with my friends, I understood the topic more easily.” In addition, students stated that they were able to express themselves more during this process, which led to increased participation in the lesson. S5 expressed his/her opinion on the subject as follows: “In previous lessons, the teacher usually explained and we just listened. But in these lessons, we talked a lot. (…) When I share my own ideas, I feel kind of free. Each group does different things.” S6 expressed his/her opinion as follows: “It was really nice to share our ideas. Even the ones who usually speak less talked. I think being in a group gave them courage. The classroom was very lively. Everyone was talking. It felt like everyone was having fun.” On the other hand, another notable finding is that students expressed a strong desire for the differentiated instruction process not to be limited to just this unit, but to be applied to other topics in science lessons and even in other school subjects as well. Students stated that this approach made learning easier and increased their interest in the lessons. In this regard, S3 stated, “I think it should be like this for every topic, because more things stay in my mind” and S1 stated, “I would like it to be applied to other topics too, because these kinds of lessons are really nice. I don’t get bored.”

When the interview data are evaluated in terms of academic achievement, social and basic skills, it is seen that the students emphasized an increase in cooperation and helping each other. S1 expressed this situation as follows: “There were some things I didn’t understand, but my friend helped me. Then I was able to do it.” In addition, some students stated that they became more successful in science lessons after the implementation process and emphasized skills such as creativity, time management and planning. S3 stated that his/her academic achievement increased by saying, “I understood the topics better. I also got more answers correct on the exam” while S6 emphasized the positive impact of the implementation process on meaningful learning and creativity by saying, “I don’t just memorize the topic; I truly understand it. Our creativity is improving.” These findings indicate that differentiated instruction has positive effects not only on academic achievement but also on social and individual skills.

When the interview data are examined in terms of attitude and motivation toward the lesson, it is understood that students liked the science lesson more than before. The vast majority of the students stated that they developed more positive feelings toward science lessons and became more interested in lessons. S4 expressed his/her opinion on the subject as follows: “The science lesson became more exciting. Before, we mostly just listened; now we also do activities ourselves.” S6 shared his/her thoughts with the following words: “Science has now become my favourite subject (…) This lesson is both fun and thought-provoking. It would be perfect if it were like this in every unit.” 

Another category identified in the interviews is the difficulties encountered during the process and their solutions. Some students stated that they faced difficulties during the activities, but overcame these difficulties with the support of their friends or the teacher. S2 expressed this situation as follows: “I struggled a bit with some activities. It became easier when the teacher showed us (…) Now I’m not afraid in class anymore. I feel happier because my friends are always by my side.” Students who had difficulties with time management stated that they tried to resolve this issue by making plans as expressed by S5, “I panicked when I didn’t have enough time for some activities. Later, I solved it by making a plan.” These findings indicate that students tended to find solutions to the problems they encountered during the learning process.

When the data obtained from the interviews are generally evaluated, it is seen that students found the applications based on differentiated science instruction enjoyable, which enabled them to learn more easily and retain knowledge more permanently and they also developed positive attitudes and skills such as cooperation and creativity, liking the science lesson and expressing their ideas within groups.

## 4. Discussion

The main purpose of the current study is to determine the academic achievement of third-grade primary school students in the “Electrical Devices and Tools” unit and to identify students’ opinions regarding the differentiated instruction process. In this context, the impact of lesson content prepared with student-centred approaches on students’ science achievement was examined, and students’ opinions on the differentiated instruction approach were also evaluated. When the findings are evaluated as a whole, statistically significant differences in favour of the experimental group were observed, indicating that the activities carried out in line with the differentiated instruction plan had a greater impact compared to the control group, which received instruction within the framework of the standard curriculum. In addition, students’ opinions about the process also indicate that the implementation contributed positively to their learning.

As a result of the analyses conducted according to the sub-problems, while no significant difference was found between the experimental and control groups’ academic achievement regarding the “Electrical Devices and Tools” topic before the implementation, the emergence of a statistically significant difference in favour of the experimental group after the implementation indicates that differentiated instruction was effective. A review of the relevant literature reveals that the differentiated instruction approach has had positive effects on academic achievement in the topic of electricity among 6th-grade students (Avcı et al. 2022). In this respect, it can be seen that the findings of the current study are parallel to those of previous research. Due to the abstract nature of concepts related to electricity, it is well known that teaching this subject, especially at the primary education level, presents certain challenges (Carlton 1999; Kalogiannakis et al. 2018). Indeed, a study conducted on 5th-grade students found that the concept of electric current could not be distinguished from other fundamental concepts such as electric energy, electricity and electric circuits (Ntourou et al. 2021). These findings highlight that teaching science subjects accurately and meaningfully at the primary education level is critically important for students’ conceptual development. In the current study, the differentiated instruction helped overcome such challenges; students reported that they were able to correct their misconceptions more easily and learned electricity-related concepts in a more concrete, meaningful and memorable way. For example, one student stated that they better understood what to do in the event of a fire caused by electricity through the various activities conducted and learned the correct actions by discussing them with his/her peers. Another student mentioned that, through the engaging activities, they learned and remembered better how to use electricity efficiently. These statements indicate that electricity-related concepts did not remain mere memorized information but became relevant and applicable knowledge for students. On the other hand, it has been determined in the literature that differentiated instruction practices support academic achievement (Prast et al. 2018; Stager 2007; Yaprakgül 2019). 

In student opinions comparing differentiated science teaching with traditional science lessons, the most prominent elements were found to be “group work” and “learning through enjoyment.” Students expressed that, in this approach, they participated more actively in the lesson, found the process more enjoyable, and that the retention of learning increased. Patrick et al. (2009) stated that when the learning environment is enjoyable and engaging, students participate more in the lesson and their learning motivation. Similarly, Baines et al. (2016) found that collaborative group work supports students’ cognitive as well as social skills. Therefore, the findings indicate that differentiated instruction makes science lessons less boring and difficult for students and learning more meaningful and engaging.

Students stated that differentiated instruction contributed to the development of various skills such as group work, collaboration, exchanging ideas when facing difficulties, mutual assistance, self-expression and creativity. Similarly, in the study conducted by Avcı et al. (2022), it was shown that differentiated instruction brought about positive developments in students’ entrepreneurship skills, including areas such as communication, self-confidence and creativity. It can be said that this is closely related to group work and collaborative activities, which are an important part of the method. Many studies in the literature report that collaborative group work enhances students’ communication and social skills and that peer interaction strengthens their self-expression and cooperative behaviours (Gillies 2016; Johnson and Johnson 1987). Moreover, Sawyer and Henriksen (2024) emphasizes that creative thinking often emerges not individually but within group dynamics. Furthermore, it should also be noted that differentiated instruction is not solely based on group work; through its sensitivity to differences and flexible practices, it provides processes tailored to students’ individual characteristics. Individualizing the process contributes to students feeling more competent and reduces their sense of failure. This, in turn, enables them to express themselves more comfortably.

Students’ use of their strengths in the learning process is one of the fundamental principles of differentiated instruction (Tomlinson 2014). However, the effective use of these strengths is only possible when students become aware of them (Flavell 1979). Activities such as argumentation, problem-solving and product creation in the current study allowed students to discover their own strengths. In addition, learning which strategies helped them succeed during this process through experiences supported their metacognitive awareness (Veenman et al. 2006).

On the other hand, the “Türkiye Century Maarif Model (TCMM)”, which was implemented along with the updated curricula in Türkiye, adopts a differentiated instruction model that considers individual differences and is based on an inclusive classroom community, avoiding competitive and segregative approaches. The TCMM adopts a student-centred educational approach that supports flexible learning pathways. This model encompasses a holistic approach that not only focuses on students’ academic achievement but also on their skills, values and interests and thus incorporates a multifaceted assessment approach that allows individuals with different learning profiles to express their knowledge in various ways (MoNE 2024). In this context, starting from the 2024–2025 school year, the TCMM has been implemented at the 1st, 5th and 9th grade levels. When evaluated within the scope of the fifth-grade science course, it is observed that enrichment activities such as creating animations, preparing posters and designing models, addressing multiple intelligences and various areas of interest, are included as part of differentiated instruction. When evaluated within the framework of 21st century skills, these activities particularly support the development of skills such as creativity, collaboration/group work and interest/curiosity. The findings of the current study are significant in terms of showing the reflections of the individualized learning approach envisaged by the TCMM in practice. In this context, it can be said that the objectives targeted by the TCMM have been achieved in the current study.

When the outcomes of using simulations on the teaching of the topic of electricity, one of the science concepts, to 4th-grade primary school students were examined, it was found that students’ active participation made a positive contribution to their achievement (Maričić et al. 2024). The findings of the current study also support this result. In the interviews, students stated that, by means of group work, game-based activities and visual materials, their interest in the lesson increased, learning became more enjoyable and they learned the subjects in a more lasting way. The findings obtained from the interviews with the students in the current study indicate that differentiated instruction increased their active participation in the lesson. While it is seen that the teaching of the topic of electricity has been addressed with various approaches in studies involving primary school students (Anam et al. 2025; Ntourou et al. 2021; Palisbo et al. 2022; Yılmaz et al. 2020), studies conducted within the context of differentiated instruction and across different grade levels have been found to be limited (Avcı et al. 2022; Salar 2018; Uğurel 2018). 

In their evaluations of the differentiated instruction process, students expressed that they would like this approach to be applied not only to the relevant unit but also to other science topics. In the interviews, students emphasized that they participated more in the lessons through various activities, that science class became “fun” and that they remembered what they had learned in a more lasting way. This situation shows that differentiated instruction not only contributes to academic achievement but also improves students’ attitudes and motivation toward science lessons. Indeed, the literature also reports that when differentiated instruction is used in various subjects, such as different topics in science (Kaplan 2016), social studies (Akdemir 2019) or in content aimed at developing mathematical reasoning skills (Çoban 2019), it positively affects not only students’ academic achievement but also their attitudes toward the subject. 

On the other hand, studies examining the content, activities and materials available on the EIN portal (Erbay 2018) show that various other variables have also been included in research topics (Hacıoğlu 2019; Kaya 2019). In this context, it is important to evaluate the effects of differentiated instruction, which serves as the basis for the new curriculum, in relation to the lesson plans recently made accessible to teachers. Gezer and Durdu (2020), who conducted a systematic analysis of theses on EIN, stated that, based on their findings, there is a particular need for more research on EIN at the primary and high school levels. Moreover, they emphasized the need for studies examining the effects of content from various subjects available on EIN not only on students’ academic achievement but also on their motivation, attitudes and other learning variables. In this context, the current study contributes to the literature, as the findings revealed that activities based on differentiated instruction positively affected not only students’ academic achievement but also their motivation and learning processes. The study contributes to filling an important gap at the national level by not only evaluating academic achievement but also including students’ opinions. In addition, the findings show that the differentiated approach is effective in teaching abstract science concepts to students with different learning profiles and that it supports learning on an international scale as well. 

### 4.1. Limitations of the Study

An important limitation of the current study is that the implementation process was restricted to only three weeks. However, since this period coincided with the time allocated for the selected subject in the curriculum, the study was conducted within the time constraints of the existing curriculum. 

### 4.2. Practical Implications and Recommendations for Future Research

Based on the findings obtained, it is possible to highlight several important points that could guide future research. Today, under the influence of globalization, the number of students with diverse cultural and national backgrounds is increasing in many regions of our country. This situation necessitates the inclusion of cultural elements such as language, values, communication styles and learning expectations, alongside individual differences in the classroom, in the teaching process. Therefore, how differentiated instruction practices are designed in a culturally responsive manner and how they are integrated with teacher competencies and classroom management strategies should be addressed and comparative studies examining their applicability and effectiveness in different cultural contexts can be conducted.

For future studies, a small-scale, exploratory qualitative study focusing on how students experience the differentiation model applied in the current study can be suggested. Such a qualitative design can provide in-depth data to reveal students’ subjective experiences, perceptions and interpretations of the learning process, rather than causal relationships. However, this approach will particularly require the careful structuring of developmentally appropriate and non-directive data collection tools, such as interview or focus group questions. 

In the current study, instruction in the experimental and control groups was conducted by different teachers. The teachers were matched based on certain characteristics, thereby aiming to minimize the effect of teacher differences on the research outcomes. However, in future studies, having the same teacher implement both the differentiated instruction approach and traditional teaching methods could allow for a more direct comparison of the effects of the methods. 

It is known that many teachers value individual differences in classroom practices and, accordingly, adopt various teaching methods. However, these practices need to be structured in a systematic way that takes into account individual differences such as students’ learning profiles, interests, readiness levels and multiple intelligences. In this context, longitudinal studies that focus on the holistic implementation of the key components of differentiated instruction, including content, process, product and learning environment, and aim to monitor the long-term effects of these practices should be conducted. 

To enhance the effectiveness of differentiated instruction, the use of innovative technologies such as AI-supported digital platforms, augmented reality (AR) and adaptive learning software is becoming increasingly important. Such tools enable the instructional process to be planned flexibly, in a personalized and data-driven manner based on students’ individual differences. In this connection, the integration of emerging digital technologies into differentiated instruction practices can be supported through empirical research examining the effects of this integration on instructional processes and student achievement. Moreover, studies comparing the applicability of these technologies across different grade levels, as well as focusing on teachers’ attitudes toward technology, their implementation competencies and roles can also provide significant contributions. 

Finally, in order to establish a stronger foundation for future studies, the primary issue to be addressed is the updating of undergraduate teacher training programs and the increase in investments in teacher training. It is stated that in countries like Canada and the United States, teacher training programs include courses on differentiated instruction, whereas in teacher training programs in Türkiye, the emphasis on differentiated instruction is weak (Zoraloğlu and Şahin 2022). Thus, future research can seek ways of including practical examples of differentiated instruction in undergraduate teacher training courses.

## Figures and Tables

**Table 1:** Dimensions of differentiated instruction based on student characteristics and implementation examples.

Student Characteristic	Dimension of Differentiation	Implementation Examples
Readiness	Content	Texts prepared at different levels and experiments that take prior knowledge into account
Learning Style	Process	Creating mind maps, game-based activities, experiments and discussions
Area of Interest	Product	Designing posters, writing stories, and preparing videos
Emotional/Environmental Needs	Learning Environment	Silent work area, digital content corner, group work stations, U-shaped seating arrangement

**Table 2:** Experimental Design.

Group	Pre-Test	Intervention	Post-Test
Experimental	EDT-AAT *	Instructional practices based on differentiated instruction	EDT-AAT
Control	EDT-AAT	Teaching practices based on the teacher’s existing methods	EDT-AAT

* Electrical Devices and Tools-Academic Achievement Test.

**Table 3:** Information about the Teachers in the Groups.

Teachers	Age	Professional Experience	Undergraduate Program Graduated
Experimental	39	17	Primary Teaching
Control	40	17	Primary Teaching

**Table 4:** The distribution of the first-term report card grades in the 3rd-grade science course for the students in the study groups.

Group	Report Card Grade								
	Very Good		Good		Moderate		Need Improvement		N
Experimental	15	62.5%	6	25%	3	12.5%	-	-	24
Control	12	57%	7	33%	2	10%	-	-	21

**Table 5:** Differentiation process.

Week	Differentiated Element	Individual Difference Taken into Account	Technique Used
1	Content	Readiness	Tiered instruction, entry points
2	Content	Readiness	Tiered instruction
3	Content-product	Readiness–area of interest	Tiered instruction

**Table 6:** EDT-AAT Item analysis results.

Item	Item Difficulty Index (p)	Item Discrimination Index (r)	KR-20 Reliability Coefficient
I1	.52	.37	.76 ^1^
I2	.48	.70	
I3	.38	.44	
I4	.68	.48	
I5	.62	.52	
I6	.46	.63	
I7	.68	.41	
I8	.58	.41	
I9	.40	.78	
I10	.70	−.07	
I11	.52	.37	
I12	.48	.70	
I13	.38	.44	
I14	.68	.48	
I15	.62	.52	
I16	.46	.63	
I17	.68	.41	
I18	.56	.30	
I19	.40	.78	
I20	.62	.52	
I21	.46	.63	

^1^ has a negative discrimination value (−0.07).

**Table 7:** Normality Test Results for the Academic Achievement Pre-test and Post-test Data of the Study Group.

EDT-AAT	Groups	N	Shapiro-Wilk/*p* *	Skewness	Kurtosis
Pre-test	Experimental	24	.24	.07	−1.12
	Control	21	.21	−.38	−.70
Post-test	Experimental	24	.04 *	−.036	−.90
	Control	21	.22	−.042	−.021

* *p* ˂ .05.

**Table 8:** Results of the *t*-Test conducted on the pre-test scores of the experimental and control groups.

Group	N	X	Ss	t	*p*
Control	21	9.952	2.085	−0.75	0.459 *
Experimental	24	10.5	2.735		

* *p* > .05 no significant difference.

**Table 9:** Tests of between-subjects effects: Regression slope of both groups.

Source	Type III Sum of Squares	df	Mean Square	F	Sig.	Partial Eta Squared	Noncent. Parameter	Observed Power ^b^
Corrected Model	484.504 ^a^	3	161.501	26.204	.000	.657	78.611	1.000
Intercept	3.395	1	3.395	.551	.462	.013	.551	.112
Group	13.703	1	13.703	2.223	.144	.051	2.223	.308
Pre-test	362.494	1	362.494	58.815	.000	.589	58.815	1.000
Group × pre-test	4.114	1	4.114	.668	.419	.016	.668	.126
Error	252.696	41	6.163					
Total	9726.000	45						
Corrected Total	737.200	44						

^a^ R Squared = .657 (Adjusted R Squared = .632), ^b^ Computed using alpha = .05.

**Table 10:** Descriptive statistics on the achievement test.

EDT-AAT	Group	N	Mean	SD
Pre-test	Experimental	24	10.5	2.735
	Control	21	9.952	2.085
Post-test	Experimental	24	15.54	3.912
	Control	21	12.52	3.763

**Table 11:** Tests of between-subjects effects: Regression slope of both groups.

Source	Type III Sum of Squares	df	Mean Square	F	Sig.	Partial Eta Squared	Noncent. Parameter	Observed Power ^b^
Corrected Model	480.389 ^a^	2	240.195	39.283	.000	.652	78.565	1.000
Intercept	6.554	1	6.554	1.072	.306	.025	1.072	.173
Pre-test	378.386	1	378.386	61.883	.000	.596	61.883	1.000
Group	61.367	1	61.367	10.036	.003	.193	10.036	.872
Error	256.811	42	6.115					
Total	9726.000	45						
Corrected Total	737.200	44						

^a^ R Squared = .652 (Adjusted R Squared = .635), ^b^ Computed using alpha = .05.

**Table 12:** Categories, codes and frequency values obtained from the semi-structured interviews.

Theme	Category	Codes	*F*
Students’ Opinions About Differentiated Science Instruction	Learning/teaching process	Expressing your opinion	3
		Group work and collaboration	6
		Individual activity	2
		Learning from peers	5
		Permanent learning	3
		Learning with fun	6
		Learning with ease	3
		Meaningful learning	1
		Passive nature of traditional lessons	5
		Student-centred structure of the new process	3
		Request for its use in other science subjects as well	5
	Academic achievement, social and individual skills	Science achievement	2
		Creativity	1
		Time management	1
		Planning	1
		Cooperation	5
	Attitude and motivation towards the instruction	Courage and self-confidence	3
		Feeling excited	1
		Enjoying science classes	6
	Difficulties encountered/Solutions	Time/Planning	1
		Difficulty in activities/Asking for help from friends and teachers	4

## Data Availability

The original contributions presented in this study are included in the article. Further inquiries can be directed to the corresponding author.

## Abbreviations

The following abbreviations are used in this manuscript:

MoNE	Ministry of National Education
EIN	Education information network
TCMM	Türkiye century maarif model
EDT-AAT	Electrical Devices and Tools-Academic Achievement Test
